# Biological Insights into Intestinal Adaptation from Preclinical Models of Short Bowel Syndrome

**DOI:** 10.3390/cells15181642

**Published:** 2026-09-10

**Authors:** Cesare Pane, Pierluigi Puca, Miriam Di Mattia, Sara Troisi, Marianna Kashyrina, Marco Pizzoferrato, Letizia Masi, Laura Parisio, Maria Cristina De Rosa, Beatrice Scagnoli, Marcello Chieppa, Valentina Petito, Loris Riccardo Lopetuso, Franco Scaldaferri, Alfredo Papa

**Affiliations:** 1 CeMAD Translational Research Laboratories, Digestive Disease Center (CeMAD), Department of Medical and Surgical Sciences, Fondazione Policlinico Universitario Agostino Gemelli IRCCS, 00168 Rome, Italy; cesare.pane.lab@gmail.com (C.P.); sara.troisi02@icatt.it (S.T.); letizia.masi@policlinicogemelli.it (L.M.); bea.scagnoli@gmail.com (B.S.); valentina.petito@policlinicogemelli.it (V.P.); franco.scaldaferri@policlinicogemelli.it (F.S.); 2 UOC Gastroenterologia, Fondazione Policlinico Universitario Agostino Gemelli IRCCS, 00168 Rome, Italy; pierluigi.puca@unicatt.it (P.P.); marco.pizzoferrato@policlinicogemelli.it (M.P.); 3 Department of Translational Medicine and Surgery, Università Cattolica del Sacro Cuore, 00168 Rome, Italy; 4 IBD Unit, Centro per le Malattie dell’Apparato Digerente (CEMAD), Dipartimento di Scienze Mediche e Chirurgiche, Fondazione Policlinico Universitario Agostino Gemelli IRCCS, 00168 Rome, Italy; lopetusoloris@gmail.com; 5 Center for Advanced Studies and Technology (CAST), “G. D’Annunzio” University of Chieti-Pescara, 66100 Chieti, Italy; miriam.dimattia@unich.it; 6 Department of Medicine and Ageing Sciences, “G. D’Annunzio” University of Chieti-Pescara, 66100 Chieti, Italy; 7 Department of Experimental Medicine (DiMeS), University of Salento, 73100 Lecce, Italy; marianna.kashyrina@unisalento.it (M.K.); marcello.chieppa@unisalento.it (M.C.); 8 Gastroenterology Unit, Ospedale Isola Tiberina Gemelli Isola, 00186 Rome, Italy; laura.parisio.fw@fbf-isola.it; 9 Institute of Chemical Sciences and Technologies ‘‘Giulio Natta’’ (SCITEC)-CNR, 00168 Rome, Italy; mariacristina.derosa@cnr.it; 10 Department of Life Science, Health, and Health Professions, Link Campus University, 00165 Rome, Italy

**Keywords:** short bowel syndrome, crohn’s disease, tissue engineering, organoids, animal models

## Abstract

Short bowel syndrome is a rare and clinically heterogeneous condition resulting from extensive intestinal resections or functional impairment, leading to malabsorption, fluid and electrolyte losses, and potential progression to intestinal failure requiring long-term parenteral nutrition. The long-term outcome of SBS is largely determined by intestinal adaptation, a progressive physiological response involving epithelial remodeling, intestinal stem cell expansion, lineage-specific proliferation, vascular remodeling, and the activity of trophic mediators—most notably glucagon-like peptide-2 (GLP-2)—whose clinical relevance is exemplified by teduglutide. Despite significant therapeutic advances, the mechanisms underlying adaptation remain incompletely understood, and preclinical models are essential tools for addressing this gap. In vitro systems—including Caco-2 epithelial cultures, intestinal organoids and enteroids, and tissue-engineered intestinal constructs—enable pathway-specific mechanistic investigation and hold promise as regenerative platforms. Murine surgical models of small bowel resection and ileocecal resection provide an integrated in vivo context for dissecting cellular and molecular mechanisms of adaptation. Large animal models, particularly minipig platforms, offer anatomical and physiological proximity to humans required for translational and therapeutic evaluation. In this narrative review, we provide an updated overview of these preclinical systems, critically examining their respective strengths, limitations, and translational relevance to advance the understanding of intestinal adaptation and inform the development of more effective therapeutic strategies for SBS (graphical abstract).

## 1. Short Bowel Syndrome and Intestinal Failure: Clinical Framework and Rationale for Preclinical Modeling

Short Bowel Syndrome (SBS) is a digestive condition arising from extensive intestinal resections, congenital abnormalities, or functional impairment, resulting in inadequate absorption of nutrients, fluids, and electrolytes [1]. When the residual intestine cannot sustain nutritional and fluid homeostasis without intravenous support, SBS may progress to intestinal failure (IF), requiring parenteral nutrition (PN) or parenteral support [2]. Although rare, SBS represents one of the leading causes of chronic IF and carries a substantial clinical burden, particularly in patients requiring long-term PN [2,3].

**Figure 1:**
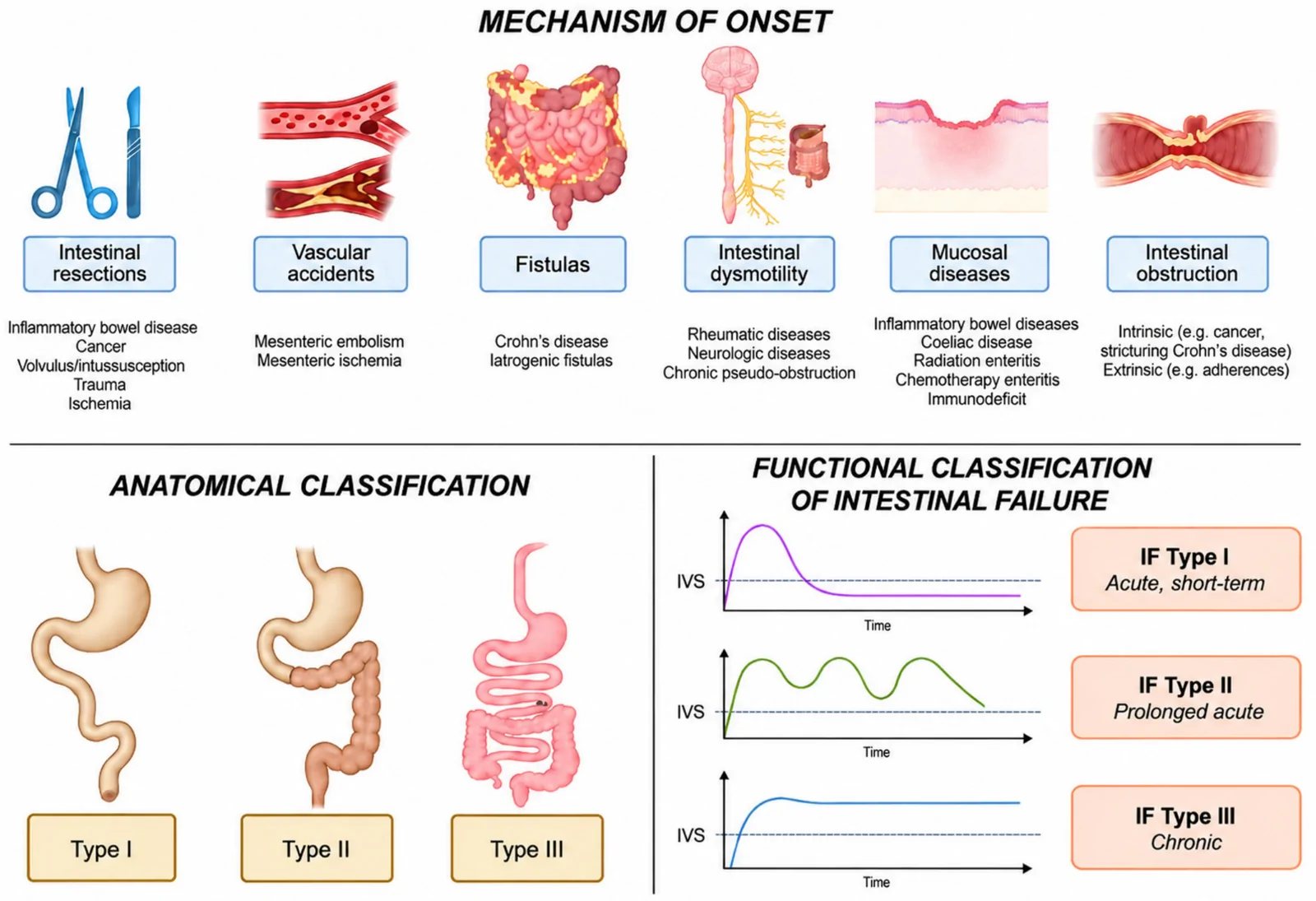
Clinical and anatomical framework of SBS and IF. The top panel illustrates major conditions leading to SBS or IF, including extensive intestinal resection, vascular events, fistulas, intestinal dysmotility, mucosal diseases, and intestinal obstruction. The bottom left depicts the main anatomical configurations of SBS: SBS Type I, end-jejunostomy; SBS Type II, jejuno-colonic anastomosis; and SBS Type III, jejuno-ileal anastomosis. The bottom right illustrates the functional classification of IF according to its clinical course and duration of intravenous supplementation requirement: IF Type I, acute and short-term; IF Type II, prolonged acute; and IF Type III, chronic.

The clinical course of SBS is highly heterogeneous, shaped by the underlying etiology—including vascular accidents, inflammatory or malignant resections, fistulas, dysmotility, mucosal disorders, and mechanical obstruction—as well as by the anatomy of the remnant bowel. Among these variables, residual bowel length, ileal preservation, and colonic continuity are the principal determinants of absorptive capacity, fluid balance, and PN dependence. Patients with end-jejunostomy carry the highest risk of long-term PN dependency, whereas jejuno-colonic or jejuno-ileal anastomoses generally allow better fluid and nutritional recovery [4]. These anatomical configurations, summarized in Figure 1, are not only central to clinical classification but also define the biological context that experimental SBS models must replicate.

Following resection, the remnant bowel undergoes intestinal adaptation, a progressive physiological response aimed at expanding absorptive surface and function. This process unfolds across three overlapping phases: an early phase marked by high intestinal losses and metabolic instability; a rehabilitation phase in which structural and functional remodeling progressively restores absorptive capacity; and a maintenance phase in which maximal adaptation is reached, after which patients either achieve enteral autonomy or remain dependent on chronic PN [4,5]. The extent of adaptation depends on residual bowel length, the intrinsically greater adaptive potential of the ileum relative to the jejunum, colonic continuity, and the presence of enteral luminal stimulation—whereas prolonged exclusive PN may paradoxically impair mucosal trophism [6,7,8,9].

The therapeutic relevance of intestinal adaptation is exemplified by teduglutide, a glucagon-like peptide-2 (GLP-2) analogue that promotes epithelial growth and enhances absorptive function, thereby reducing PN requirements in selected patients with SBS-IF. This benefit appears particularly pronounced in patients lacking colonic continuity, in whom endogenous GLP-2 secretion may be diminished [10]. Intestinal adaptation thus represents both a clinical therapeutic target and the central biological rationale for preclinical research, linking epithelial regeneration, stem cell dynamics, angiogenesis, and microbiota-mediated signaling to translational experimental models.

**Figure 2:**
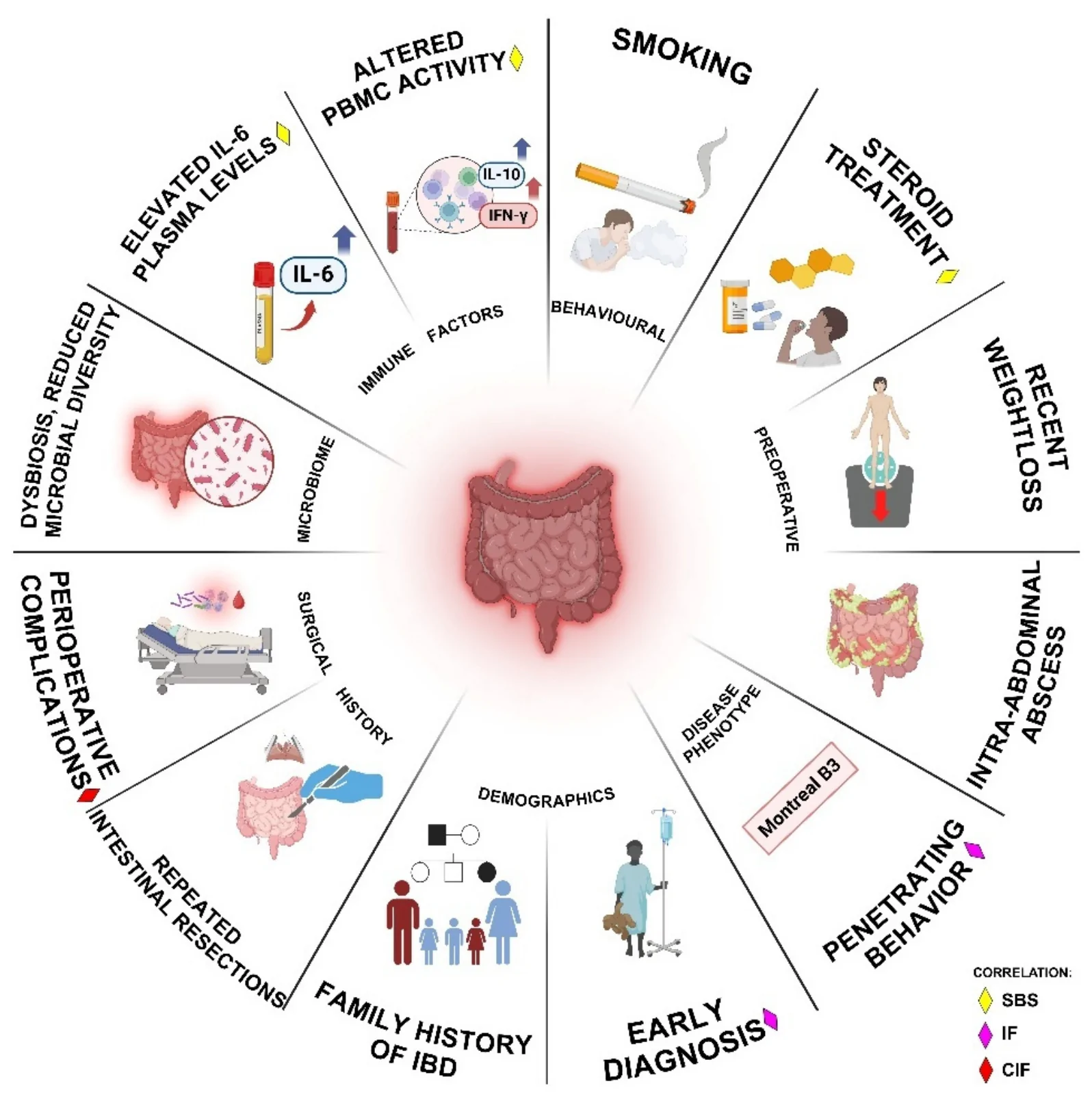
Predictor and Risk Factors for SBS, IF, and CIF in Crohn’s Disease.

Among the etiologies underlying SBS and chronic IF, Crohn’s disease (CD) holds particular clinical relevance. Although its precise epidemiological contribution is difficult to quantify, CD accounts for approximately 30–32% of patients with long-term IF in IF referral cohorts [11,12]. Multiple factors converge to increase the risk of SBS, IF, or chronic IF in CD patients, including disease phenotype, repeated surgical resections, penetrating behavior, intra-abdominal complications, steroid exposure, smoking, early age at diagnosis, immune dysregulation, and microbiome alterations [13,14,15,16,17,18]. These interacting risk factors are illustrated in Figure 2.

In this narrative review, our aim is to examine how selected preclinical models have contributed to the understanding of intestinal adaptation and regeneration in SBS and IF.

### Literature Search Strategy

This article was conceived as a narrative review supported by a structured literature search. An initial search was performed in Web of Science in May 2024 using the combinations “SBS” AND “in vitro”, “SBS” AND “stem cells”, and “SBS” AND “models”. As the records retrieved by the first two searches were encompassed by the broader “SBS” AND “models” search, the latter was used as the principal initial dataset. Retractions or retracted publications, editorial material, notes, meeting abstracts, and corrections were excluded, and retrieved records were manually screened for relevance to SBS, intestinal adaptation, and related experimental models.

During manuscript development, additional targeted searches were performed in PubMed and Web of Science to update individual sections and identify studies addressing specific experimental platforms and biological mechanisms. The search was updated before resubmission, with the final literature search performed on 20 August 2026. Relevant reference lists and citation networks of key articles and reviews were also examined to identify additional primary studies.

Original experimental studies were preferentially used to describe model characteristics and experimental findings, whereas review articles and consensus documents were used primarily to provide clinical and mechanistic context. For the in vitro and tissue-engineering component, original studies were assessed in a more structured manner to support comparative tabulation. Duplicate records, conference abstracts, non-SBS studies without direct relevance to post-resection intestinal adaptation, and articles not pertinent to the experimental platforms considered in this review were excluded.

Accordingly, this review should be regarded as a narrative synthesis rather than a systematic review.

## 2. Biological Mechanisms of Intestinal Adaptation: Rationale for Preclinical Models

Intestinal adaptation is the complex biological response that follows massive bowel resection and represents the principal determinant of long-term outcomes in SBS [19]. At the structural and epithelial level, adaptation is characterized by crypt and villus remodeling, accelerated epithelial proliferation, expansion of absorptive and secretory lineages, and progressive remodeling of the mucosal surface [20,21]. These changes are initiated and sustained by intestinal stem cells (ISCs) and their progeny, and their regulation by specific molecular pathways provides the mechanistic basis for the experimental models discussed in the following sections.

In murine models of SBS—typically involving resection of 50–75% of the small bowel or ileocecal resection—ISC expansion begins rapidly, with an early increase in side population cells within 48–72 h of surgery, followed by increased crypt fission at 7 days and sustained expansion of label-retaining cells at 6 weeks [22]. FRD1 (also known as Tis7/PC4) is induced in the intestinal epithelium during the adaptive response to small-bowel resection. Original resection studies identified growth-factor-dependent regulation of PC4/TIS7 after surgery, while experiments in Tis7-deficient mice demonstrated reduced survival and impaired postoperative recovery, with increased anastomotic inflammation and obstruction following small-bowel resection [23,24]. Wnt signaling is simultaneously upregulated and functions as a central driver of crypt hyperplasia: ApcMin mice, which carry constitutively enhanced Wnt/β-catenin signaling, display greater villus height and enterocyte proliferation 3 days after 50% proximal SBR compared with wild-type controls [25]. This pathway has also been explored in zebrafish models of SBS, where intraperitoneal administration of R-spondin 1 enhances Wnt signaling and attenuates post-resection weight loss [26]. The adaptive epithelial response involves a coordinated, lineage-specific mobilization: goblet cells and Paneth cells are activated as early as 12 h post resection and remain engaged for up to 28 days, while total enterocyte numbers increase from approximately 36 h onward, even as their relative proportion normalizes more gradually [27].

Importantly, post-resection intestinal adaptation should not be regarded solely as an epithelial proliferative response. Massive bowel resection alters tissue architecture and luminal exposure and may also modify local mechanical cues and the cellular composition of the intestinal niche, potentially engaging regulatory networks that coordinate regeneration and lineage remodeling. In this broader regenerative context, Hippo–YAP/TAZ signaling has emerged as an important mechanosensitive pathway linking changes in tissue architecture and mechanical cues with epithelial regenerative programs [28]. In addition to Wnt/β-catenin signaling, Notch and BMP pathways contribute to the regulation of intestinal epithelial lineage specification and spatial organization along the crypt axis, thereby providing additional levels of control over the balance between absorptive and secretory cell differentiation [29]. Growth-factor signaling involving EGF-family ligands and ERBB receptors, together with other mesenchyma-derived mediators, further illustrates the contribution of the stromal niche to intestinal stem-cell proliferation and epithelial regeneration; notably, mesenchyma-derived neuregulin-1 has been shown to promote intestinal stem-cell proliferation and epithelial repair [30]. Collectively, these observations indicate that intestinal adaptation is better viewed as coordinated remodeling of the epithelial compartment and its supporting niche, rather than as epithelial expansion alone. Alongside these epithelial events, early hemodynamic changes—including reduced arterial and venous blood flow and increased oxygen extraction fraction—have been documented immediately after resection in murine models and may represent a vascular trigger for subsequent mucosal remodeling [31]. Beyond these early hemodynamic changes, the vascular compartment also undergoes remodeling during post-resection adaptation. Experimental inhibition of vascular endothelial growth factor (VEGF) has been shown to attenuate the adaptive response after small-bowel resection, whereas restoration of VEGF-dependent angiogenic signaling supports mucosal and microvascular remodeling [32]. The intestinal lymphatic compartment is also affected by bowel resection. In murine models, partial small-bowel resection has been associated with persistent alterations in intestinal lymphatic morphology and impaired lymphatic transport, together with reduced lipid absorption [33]. These observations indicate that the post-resection response extends beyond epithelial remodeling to involve both blood-vascular and lymphatic compartments, although the specific contribution of lymphatic remodeling to functional intestinal adaptation remains less completely characterized. The translational relevance of these adaptive mechanisms has been further illuminated by studies using xenotransplanted human intestinal organoids in murine SBS models, which identified upregulation of SLC39A4 and SLC39A5—zinc transporter genes—as adaptive signatures in human intestinal tissue; zinc supplementation in this context promoted crypt cell proliferation and improved survival in SBS mice [34]. This finding illustrates how organoid-based and xenotransplantation approaches can uncover pathway-specific adaptive responses that may not be apparent in standard rodent models alone.

Among the humoral regulators of intestinal adaptation, growth hormone (GH) and insulin-like growth factor-1 (IGF-1) have received considerable experimental attention. In short-bowel rats maintained on PN, administration of recombinant human GH, glutamine, or their combination improved mucosal architecture—including villus height, crypt depth, and villus surface area—with the greatest benefit observed when both were co-administered [35]. Despite these encouraging preclinical results, clinical translation has remained limited: trials of GH administration in SBS patients have produced inconsistent and short-lived effects, with mucosal benefits often returning to baseline after treatment withdrawal [21]. IGF-1 similarly promotes mucosal hyperplasia and enterocyte proliferation after bowel resection in parenterally fed rodent models [36], and also supports intestinal epithelial regeneration in injury contexts through an IGF-1/mTORC1 axis involving pericryptal mesenchymal signaling and facultative stem cell activation [37]. The gap between robust animal data and limited clinical translation for both GH and IGF-1 underscores the importance of carefully designed preclinical systems in distinguishing physiological adaptation from therapeutically exploitable responses.

Beyond these trophic responses, post-resection adaptation also involves nutrient-sensitive and systemic metabolic mechanisms. Enteral nutrients act not only as substrates but also as biological signals, as illustrated by the nutrient-dependent regulation of enteroendocrine mediators such as GLP-2 [38]. At the intracellular level, mTORC1 signaling has also been directly implicated in the response to bowel resection: in murine models of 50% proximal SBR, systemic mTORC1 inhibition attenuated structural adaptation and crypt-cell proliferation, whereas forced epithelial mTORC1 activation enhanced adaptive villus growth and crypt proliferation [39]. Bile-acid signaling provides an additional link between residual intestinal anatomy and systemic metabolic regulation. In a porcine SBS model, 75% small-bowel resection was associated with bile-acid dysmetabolism and a blunted intestinal FXR activation response [40]. Because ileal FXR signaling physiologically regulates the endocrine mediator FGF19, loss of ileal mass may disrupt this enterohepatic axis; consistently, circulating FGF19 concentrations were reduced in pediatric IF and correlated with the length of the remaining ileum [41]. However, the specific contribution of the FXR–FGF19 axis to structural post-resection adaptation remains incompletely defined. At the cellular level, adaptation also involves functional and metabolic reprogramming of the surviving epithelium: following proximal small-bowel resection, distal enterocytes acquire a more proximal-like transcriptional phenotype, including increased expression of genes involved in nutrient and lipid processing [42].

Within this regulatory landscape, GLP-2 has emerged as the most therapeutically relevant mediator of intestinal adaptation. GLP-2 is a 33-amino-acid peptide derived from proglucagon and secreted by enteroendocrine L-cells of the distal small intestine and colon in response to luminal nutrients, short-chain fatty acids, inflammatory signals, and neuronal mediators [38]. Despite its short half-life due to rapid inactivation by dipeptidyl peptidase-IV (DPP-IV), GLP-2 exerts potent intestinotrophic effects—promoting crypt cell proliferation, increasing microvillus length, reducing apoptosis, enhancing barrier function, and augmenting mesenteric blood flow—through receptors expressed on subepithelial myofibroblasts, enteric neurons, and enteroendocrine cell subsets [38]. GLP-2 secretion follows a circadian pattern and is modulated by the composition and timing of enteral intake, establishing a direct mechanistic link between luminal stimulation and adaptive epithelial responses. The clinical use of teduglutide, a DPP-IV-resistant GLP-2 analogue that reduces PN requirements in patients with SBS-IF, further supports the translational relevance of the GLP-2 axis as both a central mechanism of intestinal adaptation and a primary pharmacological target for preclinical investigation [38,43]. These observations further emphasize the multicellular nature of intestinal adaptation. The enteric nervous system contributes to intestinal signaling through complex neural and neuroendocrine mechanisms [44]. Subepithelial mesenchymal cells also provide paracrine signals within the intestinal regenerative niche [30]. This concept is particularly evident for GLP-2, whose intestinotrophic effects are mediated predominantly through non-epithelial GLP-2R-expressing populations, including enteric neurons and subepithelial myofibroblasts [38]. Immune cells may also participate in the intestinal regenerative niche, although their specific contribution to post-resection adaptation remains less completely characterized [30]. The intestinal microbiota provides an additional regulatory layer: small-bowel resection can induce time- and site-dependent changes in microbial community composition [45], while microbial metabolites such as short-chain fatty acids (SCFAs) represent luminal signals capable of stimulating enteroendocrine responses, including GLP-2 secretion [38]. Together, these observations support the concept that epithelial, mesenchymal, neural, immune, and microbial components interact within the adaptive intestinal environment rather than acting as isolated pathways.

The pathways and processes described above—including ISC activation, lineage-specific epithelial remodeling, Wnt and Hippo–YAP/TAZ signaling, Notch/BMP-dependent lineage regulation, stromal niche and growth-factor interactions, vascular and lymphatic remodeling, trophic hormone responses, nutrient-sensitive and metabolic signaling, bile acid–FXR–FGF19 regulation, GLP-2-mediated intestinotrophic effects, and neural, immune, and microbiota-associated signaling—collectively define the integrated biological landscape of post-resection intestinal adaptation. Conceptually, these responses can be viewed as a continuum in which resection-associated anatomical, mechanical, luminal, and metabolic stimuli remodel the intestinal niche, drive tissue-level adaptation, and ultimately contribute to functional recovery and reduced dependence on parenteral support. Importantly, no single preclinical model is expected to reproduce all these components, and individual platforms should therefore be interpreted according to the specific biological mechanism or translational question being addressed. A summary of these adaptive mechanisms and their experimental contexts is provided in Table 1. The following chapters examine how in vitro systems and in vivo surgical models recapitulate selected components of this adaptive response, and to what extent each platform can serve as a valid tool for biological investigation and therapeutic discovery in SBS.

## 3. In Vitro Models of SBS

In vitro models offer a controlled experimental framework for investigating the cellular and molecular mechanisms of intestinal adaptation described in the preceding section. By isolating specific components of the adaptive response—including intestinal stem cell (ISC) proliferation and differentiation, epithelial lineage remodeling, hormonal signaling through GLP-2 and IGF-1, and niche-dependent regulatory interactions—these systems allow mechanistic questions to be addressed with a precision that is difficult to achieve in vivo. The following sections review the principal in vitro platforms currently employed in SBS research, from two-dimensional epithelial cultures and three-dimensional organoid models to tissue-engineered intestinal constructs designed for potential therapeutic applications.

### 3.1. Cellular and Organoid Models of Intestinal Adaptation

Caco-2 cells, a human colorectal adenocarcinoma-derived epithelial cell line, have been widely used as a simplified in vitro system to investigate selected aspects of intestinal epithelial proliferation, cell-cycle regulation, and differentiation, although Caco-2 cultures actually do not reproduce the multicellular, structural, and systemic features of SBS and should therefore not be considered a disease-specific model of intestinal adaptation. However, early in vitro studies reported that GLP-2 increased Caco-2 cell proliferation and implicated MAPK and PI3K signaling in this response [34]. Similarly, the GLP-2 analog teduglutide was shown to promote Caco-2 proliferation and cell-cycle progression while reducing the expression of several differentiation markers, including GLUT2, DPP-4, villin, and sucrase-isomaltase [35].

These findings should nevertheless be interpreted cautiously in relation to the physiological mechanism of GLP-2 action. The expression and functional relevance of GLP-2R in mature intestinal epithelial cells remain uncertain, and substantial evidence indicates that the intestinotrophic effects of GLP-2 in vivo are predominantly mediated indirectly through GLP-2R-expressing non-epithelial cells, including enteric neurons and subepithelial mesenchymal cells, and downstream paracrine mediators such as IGF-1 [46,47]. Moreover, Caco-2, as cancer-derived cells, have altered cell-cycle regulation, metabolism, and differentiation, representing additional limitations when using this system to model normal intestinal epithelial biology.

Recent advances have increasingly exploited intestinal organoid-based systems to investigate epithelial homeostasis, regeneration, and intestinal adaptation. However, the term “organoid” encompasses distinct experimental platforms that differ in their cellular origin and complexity. Adult intestinal stem cell-derived cultures from the small intestine are commonly referred to as enteroids (and as colonoids when derived from the colon) and primarily reproduce the epithelial compartment, including intestinal stem cells and differentiated epithelial lineages. In contrast, human intestinal organoids (HIOs) derived from embryonic or induced pluripotent stem cells (IPSCs) recapitulate aspects of intestinal development and may contain both epithelial and mesenchymal components. Additional experimental configurations include organoid units, which consist of multicellular intestinal tissue fragments used for tissue-engineering applications, and organoid-derived epithelial monolayers, which provide experimental access to the apical and basolateral epithelial compartments [48]. These organoids closely replicate the in vivo intestinal environment, preserving natural morphology, cellular polarity, and cell-to-cell interactions [49,50,51,52].

Compared with traditional two-dimensional cell lines, intestinal organoids more closely reproduce epithelial architecture, cellular polarity, cell-to-cell interactions, and major epithelial lineages, including enterocytes, goblet cells, Paneth cells, enteroendocrine cells, and intestinal stem cells. Although avian intestinal organoids are not direct models of human SBS, the avian study was included as a comparative example demonstrating the cross-species applicability of crypt-derived organoid culture systems [39]. No human SBS-specific conclusions were drawn from this study.

Nevertheless, they should not be considered complete replicas of the native intestine [53]. Their cellular composition and differentiation state are influenced by the tissue or stem-cell source, culture medium, extracellular matrix, passage, and differentiation conditions. Moreover, conventional in vitro organoid systems lack or incompletely reproduce several components of the intestinal microenvironment, including vascular and immune networks, enteric innervation, mechanical and luminal-flow stimuli, and a stable resident microbiota. These limitations should be considered when extrapolating organoid-derived findings to the multicellular and systemic adaptive response occurring in SBS in vivo.

The culture conditions for organoids typically involve embedding stem cells within an extracellular matrix (ECM), commonly Matrigel, and supplementing growth media with essential factors that target critical signaling pathways. Wnt signaling, for instance, is indispensable for ISC proliferation and differentiation toward Paneth cells [54], whereas Notch signalling controls the balance between absorptive enterocytes and secretory cell lineages [55]. Other growth factors, such as epidermal growth factor (EGF) and bone morphogenetic protein (BMP), significantly contribute to ISC mitosis and villus formation, respectively [56,57,58]. Furthermore, the differentiation capacity and complexity of enteroid cultures can be modulated by incorporating native intestinal cellular components and microbiota. For example, the addition of macrophages, myofibroblasts, or probiotic bacteria, such as Lactobacillus rhamnosus, significantly enhances organoid proliferation and differentiation, particularly by increasing Paneth cell populations and stimulating angiogenic and anti-inflammatory responses [59].

Beyond structural complexity, intestinal organoids have been crucial for understanding stem cell biology and regenerative mechanisms in SBS. For instance, biopsies from SBS patients demonstrate increased enteroid-forming capacity compared to healthy controls, a phenomenon linked to reduced BMP signalling mediated by subepithelial myofibroblasts unique to the SBS intestinal niche [60]. Such findings highlight the pivotal role of niche-stem cell interactions during intestinal regeneration. Organoid cultures have further clarified the biological functions of gastrointestinal hormones and nutrients essential to intestinal adaptation. Growth hormone (GH) directly stimulates ISC activity and enhances crypt-organoid formation, while glutamine promotes differentiation toward goblet and enteroendocrine cells, thereby strengthening barrier integrity [61,62].

Experimental mouse studies indicate that IGF-1 is an important mediator of the intestinotrophic effects of GLP-2. GLP-2 increases intestinal IGF-1 expression and secretion, while loss of IGF-1 or deletion of the intestinal epithelial IGF-1 receptor markedly attenuates GLP-2-induced crypt-cell proliferation and intestinal growth [63]. These findings support an indirect GLP-2–IGF-1/IGF-1R axis in vivo, but do not establish an organoid-specific mechanism.

These organoid-based insights into ISC biology, niche interactions, and trophic factor responses have in turn provided the biological and cellular foundation for the development of tissue-engineered intestinal constructs, as discussed in the following section.

### 3.2. Tissue-Engineered Intestine (TEI): Development, Applications, and Transplantation

Advancements in organoid cultures have driven the development of Tissue-Engineered Intestine (TEI), a promising approach aimed at regenerating or replacing lost intestinal tissue in SBS. TEI typically involves seeding intestinal organoids derived from patient biopsies, intestinal crypts, or stem cells (human embryonic stem cells or induced pluripotent stem cells) onto biocompatible scaffolds, producing functional intestinal constructs suitable for therapeutic use. Ideally, these constructs mimic the native intestinal tissue organization, including the appropriate proportions of major epithelial cell types, and provide immunological compatibility and functional integration post transplantation [64,65,66,67]. A variety of biomaterial scaffolds have been explored, among which poly(lactic-co-glycolic acid) (PLGA) scaffolds coated with polylactic acid (PLLA) are particularly well characterized and widely used. PLGA/PLLA scaffolds exhibit optimal pore size, mechanical strength, and degradation rates, thereby providing an architecture closely comparable to that of native intestinal tissue. Studies indicate that organoids seeded on PLGA scaffolds differentiate effectively into mature intestinal tissue, successfully integrating in vivo with excellent functional outcomes, including nutrient absorption capabilities [68]. To maximise tissue generation, an optimal seeding density of approximately 20 enteroids/mm^3^ is recommended, balancing sufficient tissue formation with potential limitations on proliferation or differentiation [69].

Additional promising scaffold options include poly-glycerol sebacate (PGS), favoured for its ease of surgical manipulation and excellent biocompatibility, and polyethene vinyl acetate (PEVA). However, while PGS demonstrated successful integration and preserved intestinal microarchitecture in vivo, PEVA showed inferior integration capabilities, highlighting the critical role of scaffold composition [70,71].

Decellularized intestinal matrices derived from porcine or human tissues represent another necessary scaffold type, as they preserve essential extracellular matrix components and signaling molecules required for cell differentiation, vascularization, and overall tissue function. Such scaffolds successfully maintained their differentiation and integration capacity in animal models, resulting in effective nutrient uptake, digestion, and barrier competence post implantation [72,73,74,75,76,77]. A novel composite bioscaffold combining synthetic oxidised polyvinyl alcohol (OxPVA) hydrogel with decellularised intestinal matrix has further expanded the possibilities for integrating the advantageous mechanical properties of synthetic scaffolds with biological cues from native matrices, thereby enhancing biocompatibility and integration [78].

Despite the predominance of organoid-based TEI, alternative cellular sources have been investigated for intestinal regeneration, including IEC-6 cell lines, gastric smooth muscle cells, bone marrow-derived mesenchymal stem cells (MSCs), adipose-derived MSCs, and human embryonic stem cells (hESCs). While enteroids remain the most thoroughly investigated model, alternative cells have provided valuable insights into their potential, particularly regarding differentiation capabilities and therapeutic feasibility in various scaffold environments [66,67,79,80].

Notably, MSCs seeded onto collagen scaffolds induced transient muscular regeneration, as indicated by the temporary presence of α-smooth muscle actin-positive cells; however, stable muscle layer regeneration remains challenging [81]. Recent progress has demonstrated engineered intestinal muscle patches capable of exhibiting coordinated contractility patterns in vitro, representing substantial progress toward functional intestinal peristalsis [71].

Following in vitro TEI preparation, successful in vivo implantation is crucial for assessing tissue integration, functionality, and immunological compatibility. Omental transplantation has become the predominant surgical implantation approach, yielding generally positive outcomes regarding tissue survival, differentiation, and intestinal regeneration. Nonetheless, surgical methods have a significant influence on graft integration. For instance, Thiry–Vella loop techniques have demonstrated superior results with fewer complications and enhanced scaffold incorporation compared to more complex, multi-stage surgical procedures [70,71]. Furthermore, adjunctive therapeutic strategies, such as teduglutide administration, which is approved for the clinical treatment of IF, have shown promising results in promoting in vivo graft survival and functional integration, underscoring potential combined therapy options [77].

Innovative techniques have been explored to improve transplantation outcomes and monitoring. For example, the creation of an optical window through a dorsal skin flap chamber enabled real-time observation of engrafted, green-fluorescent protein (GFP)-labelled cells on implanted scaffolds, providing valuable data for optimising surgical techniques and scaffold integration processes [71]. Beyond traditional sites like the omentum and intraperitoneal cavity, alternative implantation sites, such as subcutaneous, renal capsule, and neck regions, have demonstrated successful outcomes, further expanding potential implantation strategies [66,67,69,76,82,83]. Table 2A,B summarize the principal in vivo applications of organoid-based TEI and alternative cell- and scaffold-based intestinal tissue-engineering approaches, respectively. Importantly, the endpoints reported across studies reflect different levels of evidence, ranging from cell survival, histological tissue formation and epithelial differentiation to vascularization, innervation, absorptive or barrier function, and contractility. Therefore, evidence of tissue formation or marker expression should not be interpreted per se as demonstration of functional intestinal integration or replacement. Table 2A,B therefore report the specific endpoints evaluated in each study, allowing the level of evidence supporting structural or functional integration to be distinguished.

Although TEI research has made significant progress, limitations persist. Current engineered constructs often lack critical components of the native intestine, notably fully developed mesenchymal, neural, and immune elements required for complete functionality. Future studies will need to address these gaps, refining scaffold compositions, cellular sources, and culture conditions to enhance TEI’s clinical applicability and efficacy. Crucially, the translation of in vitro findings into therapeutically meaningful outcomes depends on their validation in physiologically integrated systems. In vivo surgical models—ranging from murine short bowel resection models to large animal platforms—provide the complex anatomical, hormonal, and microbial environment that in vitro systems cannot recapitulate, and represent the necessary next step in the experimental continuum reviewed in the following chapter.

## 4. In Vivo Models of SBS

While in vitro systems and tissue-engineered constructs have substantially advanced our mechanistic understanding of intestinal adaptation, they cannot fully replicate the anatomical complexity, hormonal milieu, microbial environment, and systemic physiology that characterize SBS in vivo. Animal models bridge this gap by providing an integrated biological context in which adaptive responses, regenerative strategies, and pharmacological interventions can be evaluated under conditions that more closely approximate the human disease. Murine models have been instrumental in dissecting the cellular and molecular mechanisms of adaptation, whereas large animal models—particularly minipig platforms—offer the anatomical and physiological proximity to humans required for translational and preclinical therapeutic studies.

### 4.1. Murine Models of SBS: Surgical Techniques and Adaptation Mechanisms

Murine models of SBS typically involve massive intestinal resections designed to replicate human surgical scenarios. Two well-established models include the 50% proximal small bowel resection (SBR) and distal ileocecal resection (ICR). In the 50% SBR model, approximately 15 cm of jejunum is removed, whereas the ICR model involves resection of 12 cm of ileum, cecum, and proximal colon, which better represents neonatal SBS [22,27,92]. Recently, selective vascular approaches further refined surgical techniques, significantly improving survival rates [93]. In mice, intestinal continuity is restored with an end-to-end anastomosis using optimal suture sizes, particularly 9-0, which significantly reduces morbidity [94]. Prevention of postoperative obstruction involves initiating liquid diets two days before surgery, with solid food reintroduced after one week [22]. Postoperatively, adaptive processes occur rapidly, peaking within three days, characterized by increased crypt depth, villus height, and mucosal surface area [27,94]. Intestinal stem cells (ISCs) play a central role in this adaptation. Histological analysis after ICR shows significant crypt expansion within the first week, driven by enhanced crypt fission and proliferation at the crypt base, as evidenced by increased BrdU labelling. Crypt depth and villus height initially surge, eventually normalising by 16 weeks, although intestinal circumference continues to expand for months [22].

Intestinal adaptation also involves the rapid expansion of secretory lineages, notably goblet and Paneth cells, which are critical for maintaining barrier function and antimicrobial defense. Goblet cells increase significantly as early as 12 h post resection, whereas Paneth cell numbers expand rapidly but temporarily move upward in crypts before returning to basal positions [22]. Interestingly, early expansions of these lineages do not result from newly proliferated cells, suggesting alternative adaptive mechanisms [27].

An essential aspect of intestinal adaptation is the regional reprogramming of cellular function. Following proximal resections, enterocytes in the remaining distal segments acquire characteristics similar to those of the proximal segments, thereby enhancing nutrient absorption. Single-cell RNA sequencing has demonstrated increased expression of genes typically associated with proximal enterocytes (Apoa4, Apoc3, Lct, Ephx2, Fabp1) and decreased expression of distal markers (Fabp6). Transcription factors, including Creb3l3, crucial for lipid metabolism, and ADA, a villus-tip marker, are significantly upregulated and expanded throughout villus surfaces post resection, highlighting remarkable cellular plasticity [42].

Angiogenesis is another critical adaptive response following intestinal resection. Angiogenic factors, particularly VEGF, are essential for vascular expansion and tissue remodeling. Studies indicate that VEGF plays a central role in adaptation, as inhibiting VEGF or reducing its availability through sialoadenectomy (the removal of VEGF-rich salivary glands) severely impairs adaptive responses. However, combined supplementation of VEGF and EGF fully restores adaptation, confirming the critical contribution of angiogenesis to tissue regeneration and mucosal growth [32].

The role of GLP-2 in enhancing intestinal adaptation has also been extensively investigated. Due to its rapid inactivation by dipeptidyl peptidase IV (DPPIV), oral treatment with DPPIV inhibitors (e.g., sitagliptin) prolongs the action of GLP-2. This treatment significantly increases villus height, crypt depth, and proliferation while reducing apoptosis, highlighting GLP-2’s therapeutic potential in enhancing early adaptation post resection [95]. Finally, changes in intestinal microbiota following massive resections have significant adaptive implications. Although short-term microbiota changes are minimal, long-term analyses reveal substantial differences, characterised by increases in beneficial genera, such as Lactobacillus, Lachnospiraceae, and Lactococcus, accompanied by simultaneous reductions in Enterobacteriaceae. These microbiota shifts suggest potential therapeutic targets to enhance intestinal adaptation and manage complications associated with SBS [45]. Table 3 summarizes the proposed murine models of SBS.

### 4.2. Porcine Models of SBS: Therapeutic Approaches and Clinical Translation

Porcine models, due to their anatomical similarity to human gastrointestinal tracts, have become essential in translational SBS research, enabling the testing of novel therapeutic strategies and regenerative techniques. The studies reviewed below span pharmacokinetic profiling, nutritional interventions, microbiota modulation, scaffold-based tissue engineering, and growth factor therapies, reflecting the breadth of translational questions that this model system is uniquely positioned to address.

An important area of research involves the pharmacokinetics of therapeutics in SBS. SEFA-6179 (orziloben), an orally administered, structurally engineered medium-chain fatty-acid analogue, was investigated in minipigs undergoing either jejuno-ileal anastomosis (Type III) or pseudojejunostomy (Type I). Pharmacokinetic studies demonstrated substantially reduced absorption of SEFA-6179 after resection in both minipig models compared with the pre-resection state. Absorption was greater in the Type III model with colonic continuity than in the pseudojejunostomy model. These findings indicate that remnant bowel anatomy can influence oral drug exposure and may require anatomy-specific dose optimization in patients with SBS [96]. In preterm piglets with IF-associated liver disease, administration of SEFA-6179 reduced biochemical cholestasis, steatosis, bile-duct proliferation, and fibrosis [97].

Recent advances in SBS treatment have also highlighted the potential of exclusive enteral nutrition in managing neonatal piglets with SBS. In a porcine model, enteral nutrition alone, without PN, resulted in significant fat malabsorption and deficiencies in fat-soluble vitamins like vitamin D and E, revealing challenges in intestinal adaptation post resection [98]. Fecal microbial transplant has also been explored, showing a transient increase in gut microbial diversity. While the changes were short-lived, this practice was deemed safe in neonatal piglets and demonstrated potential to influence gut microbiota in SBS models, although only in a short term context [99]. Alongside nutritional and microbiological approaches, minipig models have also been employed to evaluate scaffold-based and pharmacological strategies aimed at structural intestinal regeneration.

Tissue engineering approaches using various scaffold types have also been explored in minipig models. Acellular dermal matrix grafts, despite their successful use in other regenerative applications, have shown limited effectiveness for intestinal regeneration due to significant inflammatory responses, rapid degradation, and failure to form structured neo-intestinal tissue, highlighting the need for improved scaffolding strategies [100]. Conversely, allogenic aortic grafts demonstrated encouraging outcomes. Replacement of intestinal segments with vascular grafts led to the progressive replacement of graft material by intestinal-like tissues, demonstrating promising structural integration. Microscopic analyses revealed inflammatory cell infiltration, muscle fibre formation, and lymphoid nodules that mimicked the native intestinal submucosa by 150 days post-implantation, providing preliminary evidence supporting vascular grafts as viable scaffolds for intestinal bioengineering [101]. Innovative mechanical approaches such as spring-mediated distraction enterogenesis have demonstrated promising outcomes in enhancing intestinal length. In these models, encapsulated nitinol springs implanted within intestinal segments induced substantial segmental lengthening, with length increases of approximately 60%. Importantly, this mechanical elongation preserved normal enteric endocrine function and vitamin B12 absorption capabilities, demonstrating significant potential as a clinical therapy for managing bowel length deficiency in SBS [102].

Milk Fat Globule Epidermal Growth Factor-8 (MFG-E8) has shown promise in promoting site-specific intestinal growth, particularly increasing ileum length in the jejuno-ileal anastomosis model. This supports the potential of growth factor-based therapies in SBS management [103]. Similarly, the combined administration of GLP-2 and epidermal growth factor (EGF) significantly enhanced intestinal lengthening in neonatal piglets with SBS, particularly in the jejuno-colic model, suggesting a synergistic therapeutic approach to improve intestinal structure and function post-resection [104]. Further pharmacological strategies have targeted the GLP-2 secretory axis and lipid metabolism, with variable results in terms of structural adaptation.

Takeda G protein-coupled receptor 5 (TGR5) agonists, such as ursolic acid and olive extract, have been shown to enhance GLP-2 secretion, though they did not significantly improve intestinal adaptation post resection, highlighting the complexity of intestinal regeneration in SBS [105]. A novel docosahexaenoic acid formulation (DHA-ALT^®^) improved intestinal absorption and systemic DHA levels in neonatal pigs, enhancing intestinal adaptation and suggesting it could be a valuable adjunct therapy for improving fatty acid absorption in SBS patients [106]. Finally, a novel ambulatory SBS model incorporating Total PN showed increased gut density in SBS piglets, likely due to intestinal adaptation post resection [107]. Table 4 summarizes the proposed large animal models of SBS.

### 4.3. Limitations and Future Directions of SBS Animal Models

Despite the considerable progress reviewed in this chapter, both murine and large animal models of SBS retain important limitations that must be acknowledged when interpreting experimental findings. Murine models offer unmatched accessibility for mechanistic and genetic studies, but their small body size, short lifespan, and anatomical differences from the human gastrointestinal tract—including the absence of a well-developed colon analogue in some resection configurations—limit the direct extrapolation of findings to clinical practice. Minipig models more closely approximate human intestinal physiology, bowel length, and surgical anatomy, and are therefore better suited for evaluating therapeutic strategies in a translationally relevant context; however, their use is constrained by substantial costs, technical complexity, and the limited availability of validated experimental protocols.

A broader limitation shared by all in vivo models is the difficulty of reproducing the full clinical heterogeneity of human SBS, which encompasses distinct etiologies, variable residual anatomy, different degrees of colonic continuity, and diverse comorbidities. No single animal model captures this complexity, and the selection of an appropriate platform necessarily involves trade-offs between mechanistic resolution and translational fidelity.

Looking forward, the most productive research strategies will likely involve the integrated use of complementary platforms: in vitro organoid and tissue-engineered systems to interrogate specific molecular pathways and test cellular therapies under controlled conditions; murine surgical models to define the in vivo biology of adaptation and validate pharmacological targets; and large animal models to bridge the gap to clinical application by evaluating surgical techniques, drug delivery, and regenerative constructs in a physiologically realistic setting. Advancing this translational continuum will require not only refinement of individual model systems, but also greater harmonization of experimental endpoints, resection protocols, and outcome measures across studies—a prerequisite for meaningful comparison and cumulative progress in SBS research.

The following section synthesizes the key insights emerging from this experimental landscape and outlines the principal challenges and opportunities that preclinical SBS research faces in translating biological understanding into clinical benefit.

## 5. Conclusions

Preclinical models occupy a central role in advancing our understanding of SBS, providing the experimental infrastructure through which the mechanisms of intestinal adaptation can be systematically interrogated and novel therapeutic strategies developed. As reviewed here, no single model system is sufficient to capture the full complexity of SBS. In vitro platforms—including Caco-2 cultures, intestinal organoids, and tissue-engineered intestinal constructs—enable controlled, pathway-specific investigation of epithelial remodeling, stem cell dynamics, and trophic signaling, and represent increasingly promising tools for regenerative applications. Murine surgical models remain indispensable for defining the in vivo biology of adaptation, validating molecular targets, and evaluating pharmacological interventions under integrated physiological conditions. Large animal models, particularly minipig platforms, provide the anatomical and metabolic context required to bridge mechanistic discoveries to clinically meaningful outcomes.

The complementarity of these systems is both a resource and a challenge: their productive integration requires greater harmonization of resection protocols, outcome measures, and experimental endpoints across studies. Advancing this translational continuum is a prerequisite for meaningful cumulative progress. Ultimately, the goal of preclinical SBS research is to translate biological understanding of intestinal adaptation into more targeted therapeutic strategies—reducing dependence on parenteral nutrition and improving long-term outcomes for patients with IF.

## Figures and Tables

**Table 1:** Summary of key molecular pathways and biological processes relevant to post-resection intestinal adaptation and their experimental contexts. Time points of activation or assessment, where available, and model systems are reported alongside each pathway’s role in modulating proliferation, differentiation, remodeling, and functional recovery.

Pathway/Molecule	Activation Time Point	Experimental Model	Description	Reference
**Intestinal stem cell expansion**	48–72 h post-resection; crypt fission at 7 days; sustained at 6 weeks	Murine (ICR)	Rapid ISC expansion following ileocecal resection; increased side population cells, crypt fission, and long-term label-retaining cells.	[22]
**IFRD1 (Tis7)**	48–72 h post-resection	Murine	Induced after resection; Tis7 deletion impairs postoperative adaptation and recovery.	[24]
**Wnt/β-catenin signaling**	3 days post-resection	Murine (ApcMin, 50% proximal SBR)	Constitutive Wnt/β-catenin activation increases villus height and enterocyte proliferation after SBR.	[25]
**R-spondin 1**	Post-treatment	Zebrafish (SBS model)	Enhances Wnt signaling; attenuates post-resection weight loss.	[26]
**Hippo–YAP/TAZ mechanosensing**	Not defined after SBR	Murine DSS/irradiation intestinal injury-regeneration models; organoids	Mechanosensitive YAP/TAZ signaling in smooth-muscle cells regulates WNT4-dependent signaling and contributes to epithelial regeneration after intestinal injury; its relevance to SBS is considered within the broader regenerative framework.	[28]
**Notch/BMP signaling**	Not defined after SBR	Colonic crypt homeostasis/regenerative literature	Notch and BMP signaling contribute to epithelial lineage specification and spatial organization along the crypt axis, including regulation of absorptive and secretory differentiation.	[29]
**Mesenchymal niche/ERBB signaling**	Not defined after SBR	Intestinal injury and organoid models	Mesenchymal-derived NRG1 promotes intestinal stem/progenitor-cell proliferation and epithelial regeneration through ERBB-associated signaling.	[30]
**Goblet and Paneth cell expansion**	12 h post-resection; sustained up to 28 days	Murine (50% SBR)	Early secretory lineage expansion in crypts and villi; sustained response across adaptation phases.	[27]
**Enterocyte expansion**	From ~36 h post-resection	Murine (50% SBR)	Increase in total enterocyte number from 36 h; relative proportion normalizes gradually over time.	[27]
**Early vascular/hemodynamic response**	Immediate post-resection	Murine (50% proximal SBR)	Reduced arterial and venous blood flow, increased oxygen extraction fraction; possible vascular trigger for mucosal remodeling.	[31]
**VEGF-dependent angiogenic remodeling**	3 days post-resection	Murine SBR	Reduced VEGF availability attenuates villus and crypt adaptation after SBR; VEGF supplementation improves the vascular response, whereas combined VEGF and EGF supplementation restores structural adaptation.	[32]
**Intestinal lymphatic remodeling**	Chronic post-resection assessment (~12–15 weeks; selected lipid endpoints later)	Murine (50% and 75% proximal SBR)	Chronic SBR is associated with persistent remodeling of intestinal and mesenteric lymphatics, impaired lymphatic transport, and reduced lipid absorption/transport.	[33]
**Zinc transepithelial transport (SLC39A4/SLC39A5)**	Post-resection	Xenotransplanted human intestinal organoids; murine SBS	Upregulation of zinc transporters in human SBS-associated tissue; zinc supplementation promotes crypt-cell proliferation and improves survival in SBS mice.	[34]
**Growth Hormone (GH)**	Post-treatment	Short-bowel rats (75% resection, PN)	Improves villus height, crypt depth, and mucosal surface area; synergistic effect with glutamine co-administration.	[21,35]
**IGF-1**	Post-resection; post-irradiation	Parenterally fed rodent models	Promotes mucosal hyperplasia and enterocyte proliferation after resection; supports crypt regeneration after irradiation through IGF-1/mTORC1 signaling and pericryptal mesenchymal activation.	[36,37]
**mTORC1 signaling**	2 weeks post-resection for principal epithelial adaptation endpoints	Murine (50% proximal SBR)	Systemic mTORC1 inhibition attenuates structural adaptation and crypt-cell proliferation. Forced epithelial mTORC1 activation enhances villus growth and crypt proliferation, although epithelial mTORC1 is not required for all structural adaptive responses.	[39]
**Bile acid–FXR/FGF19 signaling**	6 weeks post-SBR in piglets; cross-sectional assessment in human IF	Porcine SBS-ALD (75% SBR); pediatric IF	Porcine SBR is associated with bile-acid dysmetabolism and a blunted intestinal FXR activation response. In pediatric IF, circulating FGF19 is reduced, particularly in the absence of residual ileum, and correlates with remaining ileal length.	[40,41]
**Enterocyte metabolic reprogramming**	7 days post-resection; selected markers also assessed at 70 days	Murine (50% proximal SBR)	Distal ileal enterocytes acquire a more proximal-like transcriptional identity after proximal SBR, with increased expression of genes involved in nutrient and lipid processing; selected molecular changes persist at day 70.	[42]
**GLP-2**	Post-resection; nutrient- and SCFA-stimulated	Murine; human (teduglutide)	Intestinotrophic peptide promoting crypt proliferation, microvillus elongation, barrier function, and mesenteric blood flow; basis for teduglutide therapy in SBS-IF.	[38]
**Microbiota remodeling**	7 and 90 days post-resection	Murine (50% proximal SBR)	SBR induces long-term, site-specific changes in ileal microbial composition; significant differences between SBR and sham animals are evident at the long-term assessment, whereas short-term differences are limited.	[45]

**Table 2:** (**A**). In vivo applications of organoid-based Tissue-Engineered Intestine models. The endpoints reported across studies represent different levels of evidence primarily emerging from histological analysis and cell survival and marker analysis. (**B**) In vivo applications of alternative cell- and scaffold-based approaches for intestinal tissue engineering. Endpoints reported primarily emerged from histological evaluation, analysis of cell survival, and assessment of cellular markers.

Type of Cell	Origin	Scaffold	Host/Implantation	Timing	Endpoints/Outcomes	Ref.
(**A**)						
OUs	Rats	PLGA scaffolds 1 cm long	Rat, peritoneal cavity	4, 8, 12 w	Histological analysis revealed well-developed and well-differentiated mucosa and submucosa within the tubular scaffolds. The epithelium consisted of columnar cells and goblet cells, arranged into crypts and villi.	[84]
Enteroids	eGFP mice’s ileum	PGS scaffolds 647 µm thick 8 mm or 25 mm diameter	Mice, omentum	2, 4, 8, 12 w	Histological analysis revealed that PGS scaffolds resemble naive intestinal microarchitecture and are well-tolerated immunologically.	[71]
Colonoids	Post-natal human colon	PLGA scaffolds 3 mm long, 2 mm thick, 1.5 mm outer diameter, +5% PLLA	NOD/SCID mice, omentum	4 w	TECs form a spherical structure with a standard crypt shape, minimal mesenchyme, and differentiated absorptive and secretory cells.	[85]
Enteroids	LGR5-eGFP transgenic mice’s crypts	PLGA scaffolds 5 mm long, 2 mm thick, 3 mm internal diameter, +5% PLLA	NOD/SCID mice, peritoneal cavity	4 w	Ideal scaffold seeding density: 20 enteroids/mm^3^. Matrigel increases neomucosal surface area with crypts, villi, intestinal epithelial lineages, myofibroblasts, and SMCs.	[86]
Enteroids ISEMFs	LGR5-eGFP/ pan-eGFP transgenic mice’s crypts	PLGA scaffolds 5 mm long, 2 mm thick, 3 mm internal diameter, +5% PLLA, +ISEMFs	NOD/SCID mice, peritoneal cavity	4 w	In vitro, enteroids exerted a chemotactic effect on ISEMFs vs. Matrigel or CM alone. Enteroids can be obtained from minimal starting material, expanded ex vivo, and implanted to produce TEI.	[69]
OUs	Human ileum, or mouse full-length small intestine tissue	none	C57BL/6 mice for mouse OU implantation; NOD/SCID mice for human OU implantation	1, 2, 4 w	In both native epithelium and TESI, a crypt structure and all the differentiated intestinal cell types were identified in all samples. Both human and mouse TESI show absorptive and digestive capacity ex vivo.	[64]
OUs	Mouse small intestine, or post-natal human intestine	none	NOD/SCID mice, omentum	4 w	The survival rate of mouse OUs with no factor supplementation remains higher after 6 days in culture. Mouse and human OUs show differentiated intestinal lineages. TESI derived from long-term OUs stained positive for the membrane sodium-glucose cotransporter.	[65]
OUs	LGR5-eGFP transgenic mice	PLGA scaffolds (measures n.s.) +5% PLLA	NOD/SCID mice, omentum	4 w	Intestinal organoids are viable after the vitrification procedure. Both vitrified and snap-frozen OUs generate TEIs with markers of differentiated epithelial cells.	[87]
OUs	Post-natal human small intestine	PLGA scaffolds 3 mm long, 2 mm thick, 1.5 mm diameter, +5% PLLA	NOD/SCID mice, omentum	4 w	Human TESI revealed the presence of all four differentiated cell types of the mature human small intestine, the muscularis mucosae, and the supporting intestinal subepithelial myofibroblasts. Nerve tissue is also present.	[88]
Enteroids	Pigs	PEVA scaffolds PGS scaffolds	Piglets, omentum	From 1 to 7 w (based on clinical judgments)	Some pigs exhibit good scaffold incorporation, cell proliferation, vascularization, and epithelialization, while others show immune reaction, poor incorporation, a track of collapse, and fibrosis.	[70]
OUs	LGR5-eGFP mice	PLGA scaffolds, 4 × 4 mm, 2 mm thick, +5% PLLA	NOD/SCID mice, omentum	1, 3, 5, 7, 14 d, 4, 6 w	TESIs expand beyond the original number of implanted cells and comprise all differentiated epithelial cell types of the native small intestine, innervated muscularis, and an intact stem cell niche.	[89]
Enteroids	Dogs	PLGA scaffolds 1 cm long, 2 mm thick, 1.6 mm diameter	Dogs, omentum or subcutaneous transplantation	4 w	Allotransplanted fetal intestinal organoids generated a significant amount of intestinal neomucosa resembling native canine intestine in structure and composition.	[90]
s	Swine jejunum segment	PLGA scaffolds 3.5 cm, 2 mm thick, 0.84 cm mean diameter +5% PLLA	Autologous piglets, intraperitoneal cavity	7 w	TEI replicates native intestine architecture with a columnar epithelium, all differentiated intestinal cell types, an innervated muscularis mucosae, intestinal subepithelial myofibroblasts, and ISCs. DCAMKL-1 expression is detected at the base of the crypts.	[82]
Enteroids	LGR5-eGFP mice or normal and diseased human intestine	PLGA scaffolds 4 cm, 200 um thick	NOD/SCID mice, omentum, Dogs (underwent mucosectomy)	14, 28, 35 d	Intestinal progenitor cells differentiate into crypt-villi structures on the scaffold. Coculture with ISEMFs, macrophages, and probiotic bacteria enhances differentiation and scaffold coverage, thereby improving mucosal regeneration in the dog rectum.	[59]
OUs	Human ileum, colon, or rat jejunum, ileum, and colon crypts	none	- NOG mice, colon; - Rats at the ileocaecal junction		Xeno-transplanted human ileum OUs reconstitute villus structures in the mouse colon, SI epithelium, and their absorption-related machinery. SIC implantation at the ileocaecal junction rebuilds organ structures of the SI, including intact vasculature and innervation, villous structures, and the lacteal.	[91]
- Patient-derived enteroids - jejunal fibroblasts - HUVEC	Human crypts (from duodenum, jejunum and ileum)	Human intestinal scaffolds from pediatric patients Porcine-derived SI	NOD/SCID mice, kidney capsule or subcutaneous transplantation	7, 14 d	Grafts show full coverage of all intestinal cell types, recapitulating the native microenvironment. Absorption, digestion, and barrier competence analysis also provide positive results. The presence of CD31^+^ cells surrounding the lumens suggests neovascularization in the grafts.	[77]
HIOs	hESCs	none	NOD/SCID mice, under the left kidney capsule	4, 8, 12 w	HIO transplantation promoted increased expression of TJ proteins (CLDN-3, CLDN-15, OCLN, and TJP1/ZO-1). Transplanted HIOs exhibit a more mature morphology than those grown in vitro. Detection of villus-crypt units in 6–12-week-old transplanted HIOs.	[66]
HIOs	hESCs	-Decellularized porcine or human intestinal matrices (~6–8 inch-long segments); -PLGA scaffolds 3 mm long, 2 mm thick, 1,5 mm diameter, +5% PLLA	NOD/SCID mice, omentum	14 w	Acellular matrix alone does not instruct hESC differentiation towards an intestinal fate; HIOs reseed acellular porcine matrices in vitro but do not thrive when transplanted in vivo. HIO-seeded PGA/PLLA scaffolds thrive in vivo and develop into a tissue nearly identical to adult human intestinal tissue.	[72]
HIOs	hESCs	none	Male and female NOD/SCID mice, under the left kidney capsule	8–9 w	Host survival and transplanted HIO engraftment are almost equivalent in male and female hosts. Transplanted HIOs from both groups have similar engraftment, growth, and epithelial and mesenchymal cytodifferentiation.	[67]
(**B**)						
Naïve stem cells	Rats’ intestinal crypt cells	2 × 1 cm ABS segments Autologous BIS	Rats, antimesenteric border of the small bowel	4, 8, 12 w	Architecture and morphology are similar between the BIS and the native intestine. Normal-appearing columnar absorptive cells and secretory GCs with standard mucin staining patterns. BIS shows functional absorptive capacities.	[74]
IEC6 cell line	Rats’ intestinal crypt cells	0.5-cm^2^ disc of polycarbonate membrane	Rats, omentum, 5 cm distal to the Treitz’s ligament	3 or 7 d	Stably transfected cells survive and grow after implantation into the rats’ SI.	[79]
Gastric SMCs	Dogs	Collagen sponge	Dogs, ileum	4, 8, 12 w	Collagen sponge scaffolds seeded with autologous SMCs can promote SI regeneration. In the SMC^(+)^ group, the luminal surface is covered by a well-developed epithelial layer with numerous villi. Implanted SMCs are also seen in the lamina propria and form a smooth muscle layer.	[80]
GFP-iPSCs HUVECs	Human	Decellularized intestinal matrix (4 cm segment of proximal jejunum) from rats	Rats neck region transplantation	4 w	After heterotopic transplantation, the grafts exhibit survival and maturation of the regenerated epithelium. Systemic venous sampling and positron emission tomography confirmed glucose and fatty acids uptake in vivo.	[76]
ISCs-containing crypts	1–3 d old rat pups	PLGA scaffolds 0.5 × 1.0 cm, 2 mm thick, +5% PLLA, +Collagen (I group) +HB-EGF (II group) +CO_2_ exposure (III group)	Rats, peritoneal cavity	4 w	TEIs from all three groups have all elements of the native SI, including Paneth cells, enteroendocrine cells, absorptive enterocytes, SMCs, ISEMFs, and actively proliferating crypt cells. III group scaffold-derived TEIs have longer villi and denser crypts due to the local delivery of the trophic factor EGF.	[83]
Ad-MSCs	Human	Decellularized intestinal matrices from rats +OxPVA composite bioscaffolds	Rats, omentum	4 w	In vitro, proliferating cells on composite bioscaffolds are significantly more numerous than on OxPVA supports alone. In vivo, OxPVA bioscaffolds show proper degradation rate, no inflammatory reactions and the presence of myofibroblastic and/or smooth muscle cells, cubic/cylindrical cells with basal nuclei, and vascular structures with organized endothelium	[78]
BMMSCS	Dogs	Collagen sponge scaffold ~5 cm	Dogs, omentum	2, 4, 16 w	The regenerated intestine has only a submucosal layer at 4 w. At 16 w after surgery, the mucosal layer covers the luminal surface of the regenerated intestine. However, the αSMA^(+)^ layer soon disappeared. Only a thin muscle layer is observed just beneath the mucosal layer.	[81]
none		Decellularized ileum and mesenteric arcade from pigs (25–30 cm)	Pigs, n.s.	Up to 24 h	Implanted scaffolds show macrophages and progenitor cells’ ingress via the blood, and CD133^+^ and CD68^+^ cells in the submucosal vasculature of the ileal tissue of the decellularized scaffold. Evidence of a vascular system able to deliver O_2_ from the host deep into the scaffold.	[75]
none		Porcine-derived small intestinal submucosa 2 × 7 cm	Dogs, 40 cm distal to the ligament of Treitz	6 m	The mucosa is covered with normal epithelium. Reorganization of neural and SMCs, as well as isoperistaltic contractility during fasting, is observed through the grafted segment. The SIS-reorganized tissue contracts in response to an ACh agonist and electrical field stimulation.	[73]

Abbreviations: CLDN, Claudin; CM, Conditioned Medium; d, days; DCAMKL-1, double cortin and CaM kinase-like-1; GFP, Green Fluorescent Protein; h, hours; HIOs, Human Intestinal Organoids; HUVEC, Human umbilical vein endothelial cells; ISCs, Intestinal Stem Cells; ISEMFs, Intestinal Subepithelial Myofibroblasts; LGR5, Leucine-rich repeat-containing G-protein coupled receptor 5; m, months; OCLN, Occludin; OUs, Organoid Units; PEVA, poly(ethylene-covinyl acetate); PLGA, Poly (lactide- co-glycolide); PGS, poly(glycerol sebacate); PLLA polylactic acid; SI, Small Intestine; SIC, small intestinalized colon; SMCs, Smooth Muscle Cells; TEC, tissue-engineered colon; TEI, Tissue-Engineered Intestine; TESI, Tissue Engineered Small Intestine; TJ, Tight Junctions; w, week; ZO, Zona Occludens; ABS, Acellular Biological Scaffolds; Ach, Acethylcoline; Ad-MSCs, Adipose Mesenchymal Stem Cells; BIS, bio-artificial intestinal segment; BMMMSCs, Bone Marrow Mesenchymal Stem Cells; d, days; EGF, Epidermal Growth Factor; GFP, Green Fluorescent Protein; GCs, Goblet Cells; h, hours; hESCs, human Embryonic Stem Cells; IECs, Intestinal Epithelial cells; ISEMFs, Intestinal Subepithelial Myofibroblasts; iPSCs, induced Pluripotent Stem Cells; m, months; O_2_, Oxygen; OxPVA, Oxidized polyvinyl alcohol; PLGA, Poly (lactide- co-glycolide); PGS, poly(glycerol sebacate); PLLA polylactic acid; SI, Small Intestine; SIS, small intestinal submucosa; aSMA, alpha smooth muscle actin; SMCs, Smooth Muscle Cells; TEC, tissue-engineered colon; TEI, Tissue-Engineered Intestine.

**Table 3:** Murine Models of Intestinal Resection: key outcomes and mechanistic insights.

Strain	Sex	Age	Type of Resection	Type of Anastomosis	Preservation of Ileocecal Valve	Preservation of Colon	Perioperative Mortality	Nutrition Regimen	Number of Animals	Duration of Follow-Up	Endpoints/Outcomes	Ref.
**C57BL/6J**	M	8–12 wk	ICR	jejuno-colonic end-to-end anastomosis	Not preserved	Partially resected (proximal right colon removed)	SHAM 5% ICR 9%	Mice were switched to a liquid diet 2 days before the operation and maintained on it over the first 7 days postoperatively to avoid obstruction, after which they were switched back to regular chow.	SHAM: n = 4–8/group ICR: n = 5–8/group	2–3 days, 4–5 days, 6–7 days, 2 wk, 6 wk, and 16 wk	Following ICR, the remaining small intestine undergoes rapid calibre increase, concomitant with significant but transient increases in villus height and crypt depth. This increase is due to an increase in the total number of crypts, resulting from an increased rate of crypt fission, which is preceded by an expansion of ISCs.	[22]
**C57BL/6J**	M	Adult	50% SBR	jejunoileal end-to-end anastomosis	Preserved	Preserved	SBR 10%	Mice were switched to a liquid diet 2 days before the operation and maintained for the duration of the experiment.	SHAM: n = 5/group SBR: n = 5/group	12 h, 36 h, 7 days, and 28 days	A significant early expansion of intestinal secretory lineages—such as Paneth cells at the crypt base and goblet cells within the villi was observed as early as 12 h after SBR and persisted for up to 28 days. This sustained expansion possibly serving to amplify the signaling pathways that initiate and maintain intestinal adaptation.	[27]
**C57BL/6**	M	8–12 wk	50% SBR	jejunoileal end-to-end anastomosis	Preserved	Preserved	N/A	Mice were switched to a liquid diet 24 h before the operation, withheld the morning of surgery, and subsequently provided 24 h after surgery.	SHAM: n = 5 SBR: n = 4	7 and 70 days	The process of adaptation involves the regional reprogramming of ileal enterocytes into mature proximal enterocytes. These cells exhibit differentiation markers, characterised by the increased expression of solute and nutrient transporters typically associated with the proximal small intestine, particularly those involved in lipid metabolism.	[42]
**C57BL/6**	F	8–10 wk	50% SBR	jejunoileal end-to-end anastomosis	Preserved	Preserved	SHAM 0% SBR~36%	Mice were switched to a liquid diet 2 days before the operation. Water was provided ad libitum for the first 24 h after surgery, after which a liquid diet was provided.	SHAM: n = 5 SBR: n = 8 SBR + SAL: n = 8 SBR + SAL + EGF: n = 9 SBR + SAL + VEGF: n = 9 SBR + SAL + VEGF + EGF: n = 6 SBR + Ad-VEGF-Trap: n = 7	3 days	In mice undergoing SBR, removal of the salivary glands attenuates the adaptive response, as evidenced by reductions in villus height, crypt depth, and DNA content. However, this response is completely restored by the simultaneous administration of VEGF and EGF. These findings suggest that the adaptive response may be mediated, at least in part, by VEGF-driven angiogenesis.	[32]
**C57BL/6J**	N/A	8 wk	50% SBR	jejunoileal end-to-end anastomosis	Preserved	Preserved	<10% (no differences in survival between groups)	Mice were switched to a liquid diet 2 days before the operation and maintained for the duration of the experiment.	SHAM: n = 5 SBR: n = 6 SBR + DPP IV-I: n = 6	3 days	Compared to untreated SBR mice, oral administration of a DPPIV inhibitor significantly increased intestinal adaptation, as indicated by increased villus height, crypt depth, elevated epithelial cell proliferation, and reduced apoptosis. DPPIV inhibitor treatment also markedly elevated circulating GLP-2 levels and upregulated the mRNA expression of β-catenin and c-myc in the ileal mucosa.	[95]
**C57BL/6**	N/A	8 wk	50% SBR	jejunoileal end-to-end anastomosis	Preserved	Preserved	SHAM~11% SBR~41%	Mice were switched to a liquid diet 1 week before the operation. Water was provided ad libitum for the first 24 h after surgery, after which a liquid diet was provided.	SHAM: n = 19 SBR: n = 27	7 and 90 days	Significant alterations in the microbial composition of the ileum were observed at 90 days post SBR, characterised by an increased abundance of Lactobacillus species and a reduced presence of Enterobacteriaceae. In contrast, no significant differences in microbial composition were detected in stool, caecal or ileal contents during the short-term period (7 days), nor in long-term stool samples. Overall, SBR leads to persistent, site-specific shifts in the ileal microbiota, while the microbial communities of more distal regions of the gastrointestinal tract remain unaffected.	[45]

Abbreviations: M, male; F, female; AdVEGF-Trap, Adenoviral Vascular Endothelial Growth Factor Trap; DPPIV-I, Dipeptidyl Peptidase IV Inhibitor; EGF, Epidermal Growth Factor; GLP-2, Glucagon-like Peptide-2; h, hours; ICR, Ileocecal Resection; ISCs, Intestinal Stem Cells; mRNA, messenger Ribonucleic Acid; N/A, Not Available; n, number of animals; SAL, Salivary Gland Removal; SBR, Small Bowel Resection; SHAM, sham-operated control; VEGF, Vascular Endothelial Growth Factor; wk, week(s).

**Table 4:** Overview of Preclinical Large Animal Models Used to study SBS and related intestinal interventions.

Strain	Sex	Age	Type of Resection	Type of Anastomosis	Preservation of Ileocecal Valve	Preservation of Colon	Perioperative Mortality	Nutrition Regimen	Number of Animals	Duration of Follow-Up	Endpoints/Outcomes	Ref.
**Yucatan minipigs**	F	5–8 months	90% SBR	JI model: jejunoileal end-to-end anastomosis PJ model: end-to-side ‘pseudojejunostomy’	JI: preserved PJ: functionally bypassed	JI: Preserved PJ: The entirety of the intraperitoneal spiral colon was bypassed, with an anastomosis created to the retroperitoneal colon immediately proximal to the rectum	0%	Intravenous normal saline was administered until the morning of postoperative day 1 for 12 h during the day and discontinued overnight. Minipigs received ad libitum access to a water. Standard swine chow was offered beginning on postoperative day 1.	SBR with jejunoileal anastomosis + aqueous SEFA-6179 (n = 5) SBR with jejunoileal anastomosis + non-aqueous SEFA-6179 (n = 5) SBR with ‘pseudojejunostomy’ + aqueous SEFA-6179 (n = 5) SBR with ‘pseudojejunostomy’ + non-aqueous SEFA-6179 (n = 5)	4 days	The PJ minipigs exhibited a more severe malabsorptive phenotype than the JI minipigs. The pharmacokinetics of SEFA-6179 are influenced by bowel anatomy and drug formulation. In both models, SEFA-6179 absorption was substantially lower than in pre-resection minipigs; however, it improved with a non-aqueous formulation. These findings have important translational implications for tailoring SEFA-6179 therapy based on individual bowel anatomy in patients with SBS, and support further clinical evaluation of colonic involvement in drug absorption.	[96]
**Yorkshire pigs**	M	5–6 wk	75% SBR	jejunoileal end-to-end anastomosis	Preserved	Preserved	SBR 25% (n = 1)	The model was specifically developed to function without PN support. EN was delivered continuously at 60% of the total daily caloric intake, combined with per-oral pig chow at 40%.	SBR: n = 4 SHAM: n = 3	21 days	Animals that underwent intestinal resection exhibited significant fat malabsorption, as evidenced by a reduced CFA, together with the progressive development of deficiencies in the fat-soluble vitamins D and E. The resected animals had higher serum triglyceride concentrations and lower HDL concentrations than the control group. This model may facilitate the study of intestinal adaptation and nutritional interventions in SBS.	[98]
**Duroc piglets**	M	Neonatal (3–7 days)	75% SBR	jejuno-colic anastomosis	Not preserved	Partially resected (3 cm removed)	SBR ~8% (1 per group)	PN was administered immediately after surgery. EN commenced on day 2 at 20% of requirements.	SBR + Saline (n = 12) SBR + FMT (n = 12) nonsurgical sow-reared control (n = 6)	7 days	FMT appears to be safe and well tolerated; there was no disease-specific mortality or incidence of sepsis. FMT induced transient changes to the intestinal microbiota (5 days after treatment) but most compositional differences did not persist.	[99]
**Wuzhishan miniature pigs**	N/A	N/A	No intestinal resection was performed. The study used an intestinal elongation model where the small bowel was transected in the middle portion.	A 3-cm-long graft was interposed in continuity with the small bowel using an interrupted two-layer end-to-end anastomosis. Then a side-to-side anastomosis was performed approximately 3 to 4 cm distal to the graft.	Preserved (not part of the small-bowel -graft interposition site)	Preserved (not part of the small-bowel -graft interposition site)	0%	Animals were maintained on a liquid diet and water for the first 48 h, followed by a full diet.	donor of allogenic ADM: n = 1 SBI + allogenic ADM. n = 2 SBI + ADM. n = 2	3 months	The grafts were fully resorbed within two to three months of the surgery. Histological analysis revealed pronounced inflammation characterised by fibrinoid necrosis, as well as extensive infiltration of neutrophils and leukocytes. Notably, well-formed intestinal structures were absent throughout the study. These results highlight the need for additional strategies, such as cellular seeding, growth factor incorporation or mechanical stimulation, to improve scaffold integration and promote intestinal tissue regeneration.	[100]
**Minipigs**	F	Young	No intestinal resection was performed. The study used interposition of a 10-cm-diameter aortic graft in the small bowel.	Jejuno-jejunal side-to-side anastomosis: to exclude 30-cm-diameter bowel. Aortic-enteric end-to-end anastomosis: connecting the 10-cm aortic graft in the middle of the excluded bowel	Preserved (not part of the small-bowel -graft interposition site)	Preserved (not part of the small-bowel -graft interposition site)	~29% (n = 2)	A low-fiber diet was given at regular intervals. Water was provided without restriction.	n = 7	1 month 3 months 5 months	Macroscopic and microscopic changes occurred in the aortic graft wall, including folding of the media, colonisation by inflammatory cells and infiltration of connective tissue buds that mimicked the structure of the bowel wall. These findings support the feasibility of using vascular grafts for intestinal bioengineering and warrant further investigation into their long-term functionality and clinical translation.	[101]
**Yucatan minipigs**	F	Juvenile	No intestinal resection was performed. The study used an intestinal elongation model involving the insertion of a nitinol spring into the ileal lumen.	No anastomosis was performed. An enterotomy was created in order to insert the nitinol spring, which was then closed using sutures.	Preserved	Preserved	0%	Animals were maintained on an enteral liquid diet.	n = 7	7 days	The intraluminal nitinol spring significantly increased the length of the porcine ileum. No significant difference was observed in the average density of chromogranin-A cells in the control and spring segments. Both the control ileum and the lengthened ileum contained vitamin B12-intrinsic factor cotransporter, as well as cells that produced 5-HT. These findings suggest that this technique could be useful in treating SBS, as it promotes functional bowel lengthening without compromising intestinal integrity.	[102]
**Landrace-Large White cross piglets**	M	Neonatal (3–4 days)	75% SBR	JI model: jejunoileal anastomosis JC model: jejuno-colic anastomosis	JI: preserved JC: not preserved	JI: preserved JC: partially resected (5 cm removed)	0%	Piglets received PN immediately postoperatively. EN commenced on day 2, delivered continuously alongside PN.	JI + Saline: n = 6 JI + MFG-E8: n = 8 JC + Saline. n = 5 JC + MFG-E8: n = 5	7 days	Treatment with MFG-E8 produces site-specific trophic effects that are restricted to models with a retained ileum (JI), where treatment increases the linear growth of the ileal segment alongside upregulated of mucosal gene expression of IGF-1 and EGFR. However, the absence of a remnant ileum is common in neonatal SBS, which limits the therapeutic utility of MFG-E8 for this condition.	[103]
**Landrace-Large White cross piglets**	M	Neonatal (4 ± 2 days)	75% SBR	JI model: jejunoileal anastomosis JC model: jejuno-colic anastomosis	JI: preserved JC: not preserved	JI: preserved JC: partially resected (5 cm removed)	SHAM ~ 18% (n = 3) JI ~ 21% (n = 5) JC ~ 17% (n = 4)	Immediately after surgery, all piglets received PN providing 100% of their daily caloric requirements. On postoperative day 2 EN was initiated at 20% of daily caloric requirements.	SHAM + Saline: n = 4 SHAM + GLP-2: n = 5 SHAM + EGF-cm: n = 4 SHAM + EGF-cm + GLP-2: n = 4 JI + Saline: n = 7 JI + GLP-2: n = 5 JI + EGF-cm: n = 6 JI + EGF-cm + GLP-2: n = 6 JC + Saline: n = 5 JC + GLP-2: n = 6 JC + EGF-cm: n = 6 JC + EGF-cm + GLP-2: n = 6	7 days	Combination treatment with GLP-2 and EGF-cm promoted trophic intestinal effects and structural adaptation in both the JI and JC models. For parameters including normalised intestinal and mucosal weight and villus height, the combination therapy produced similar results to GLP-2 alone. However, both treatments produced greater effects than the saline control and/or EGF-cm alone. These findings suggest that GLP-2 is the primary driver of improvements in these parameters.	[104]
**Crossbred piglets**	N/A	Neonatal (6 days)	80% SBR	jejunoileal anastomosis	Preserved	Preserved	0%	All pigs received total PN throughout the study. Treatments were infused intragastrically and flushed with cow’s milk replacer formula to provide minimal enteral nutrition every 8 h between postoperative days 2 and 10.	SHAM + DMSO SBR + DMSO SBR + UA SBR + OE n = 6–8/group	10 days	Despite stimulating GLP-2 secretion in non-resected models, UA and OE did not increase plasma GLP-2 levels or improve intestinal adaptation following massive small bowel resection. This may indicate that higher doses or more potent TGR5 agonists are required to enhance GLP-2-mediated intestinal adaptation.	[105]
**Crossbred (Hampshire x Landrace x Duroc x Yorkshire) piglets**	M–F	Neonatal (6 days)	80% SBR	jejuno-ileal end-to-end anastomosis	Preserved	Preserved	0%	PN was initiated within 3 h postoperatively. EN was initiated 24 h after resection using a sow milk replacer formula supplemented with either DHA ethyl ester (control) or DHA-ALT^®^.	SBR + DHA: n = 5 SBR+ DHA-ALT^®^: n = 5	4 days	Compared with standard DHA, the pre-emulsified DHA preparation bypassed the need for endogenous micelle formation, resulting in reduced fecal DHA losses, greater ileal tissue incorporation, higher systemic plasma levels of DHA and eicosapentaenoic acid, and enhanced intestinal adaptation, as evidenced by increased villus height in the proximal jejunum and distal ileum.	[106]
**Pigs**	N/A	Neonatal (7–10 days)	90% SBR	jejunoileal side-to-side (functional end-to-end)	Preserved	Preserved	0%	All pigs undergoing resection received Total PN using miniaturized pumps, beginning 2 days after surgery and continuing throughout the study.	SBR: n = 6	3 wk	The ambulatory Total PN SBS model effectively replicates key clinical elements of human SBS, including hyperbilirubinemia and compensatory intestinal hypertrophy highlighting its potential for future research.	[107]

Abbreviations: M, male; F, female; ADM, Acellular Dermal matrix; CFA, Coefficient of Fat Absorption; DHA, Docosahexaenoic Acid; DHA-ALT^®^, DHA with Advanced Lipid Technology, DMSO, Dimethyl Sulfoxide, EGF- cm, Epidermal Growth Factor containing media; EGFR, Epidermal Growth Factor Receptor; EN, Enteral Nutrition; FMT, Fecal Microbial Transplant; GLP-2, Glucagon-like Peptide-2; h, hours; HADM, Human Acellular Dermal Matrix; HDL, High-Density Lipoprotein; IGF-1, Insulin-like Growth Factor 1, JI, Jejunoileal, JC, jejuno-colic; MFG-E8, Milk Fat Globule Epidermal Growth Factor-8; n, number of animals; N/A, Not Available; OE, Olive Extract; PJ, Pseudojejunostomy; PN, Parenteral Nutrition; SBS, Short Bowel Syndrome; SBI, Small Bowel Incision; SBR, Small Bowel Resection; SEFA-6179, Structurally Engineered Medium Chain Fatty Acid analogue; SHAM, sham-operated control; UA, Ursolic Acid; wk, week(s).

## Data Availability

No new data were created or analyzed in this study. Data sharing is not applicable to this article.
